# Infrared heater system for warming tropical forest understory plants and soils

**DOI:** 10.1002/ece3.3780

**Published:** 2018-01-15

**Authors:** Bruce A. Kimball, Aura M. Alonso‐Rodríguez, Molly A. Cavaleri, Sasha C. Reed, Grizelle González, Tana E. Wood

**Affiliations:** ^1^ The Greenleaf Group Phoenix AZ USA; ^2^ International Institute of Tropical Forestry USDA Forest Service Luquillo PR USA; ^3^ School of Forest Resources and Environmental Science Michigan Technological University Houghton MI USA; ^4^ U.S. Geological Survey Southwest Biological Science Center Moab UT USA; ^5^ International Institute for Tropical Forestry USDA Forest Service Río Piedras PR USA

**Keywords:** climate change, global warming, heater array, infrared warming, proportional integrative derivative control, trees

## Abstract

The response of tropical forests to global warming is one of the largest uncertainties in predicting the future carbon balance of Earth. To determine the likely effects of elevated temperatures on tropical forest understory plants and soils, as well as other ecosystems, an infrared (IR) heater system was developed to provide in situ warming for the Tropical Responses to Altered Climate Experiment (TRACE) in the Luquillo Experimental Forest in Puerto Rico. Three replicate heated 4‐m‐diameter plots were warmed to maintain a 4°C increase in understory vegetation compared to three unheated control plots, as sensed by IR thermometers. The equipment was larger than any used previously and was subjected to challenges different from those of many temperate ecosystem warming systems, including frequent power surges and outages, high humidity, heavy rains, hurricanes, saturated clayey soils, and steep slopes. The system was able to maintain the target 4.0°C increase in hourly average vegetation temperatures to within ± 0.1°C. The vegetation was heterogeneous and on a 21° slope, which decreased uniformity of the warming treatment on the plots; yet, the green leaves were fairly uniformly warmed, and there was little difference among 0–10 cm depth soil temperatures at the plot centers, edges, and midway between. Soil temperatures at the 40–50 cm depth increased about 3°C compared to the controls after a month of warming. As expected, the soil in the heated plots dried faster than that of the control plots, but the average soil moisture remained adequate for the plants. The TRACE heating system produced an adequately uniform warming precisely controlled down to at least 50‐cm soil depth, thereby creating a treatment that allows for assessing mechanistic responses of tropical plants and soil to warming, with applicability to other ecosystems. No physical obstacles to scaling the approach to taller vegetation (i.e., trees) and larger plots were observed.

## 1 INTRODUCTION

Ecosystems worldwide are responding to Earth's changing climate, with important implications for their structure and function, as well as for determining central feedbacks to future climate (IPCC, 2014). These ongoing changes and associated consequences underscore the value of understanding and forecasting the effects of climatic change, yet our knowledge of how systems will respond to further increases in temperature remains notably poor (e.g., Shao, Zeng, Sakaguchi, Monson, & Zeng, 2013). This is particularly true for tropical forested ecosystems, which sustain exceptionally high biodiversity, cycle vast amounts of carbon, and represent a key uncertainty in considering terrestrial ecosystem feedbacks to future climate (Cavaleri, Reed, Smith, & Wood, 2015; Pan et al., 2011; Wood, Cavaleri, & Reed, 2012). However, while the need for an improved understanding of warming effects on tropical forests is clear, enhancing our understanding of temperature effects on these and other ecosystems remains a significant research challenge (Cavaleri et al., 2015).

Common methods for investigating temperature controls over forest composition and processes include laboratory and greenhouse experiments, soil incubations, and studies along elevation gradients (e.g., Chen, Yu, González, Zou, & Gao, 2017). While the use of field experimental warming has been less common in forested ecosystems, it holds much promise for elucidating the consequences of increasing temperatures. The majority of forest warming experiments that currently exist have primarily been implemented in temperate forests using underground cables (Melillo et al., 2002; Pries, Castanha, Porras, & Torn, 2017) but there are other viable methods. Using infrared (IR) heaters is an approach to field warming that enables warming of the vegetation and soils under free‐air, open‐field conditions, with much less disturbance than buried cables (e.g., Jarvi & Burton, 2013). IR arrays that maintain a constant temperature rise of the heated plots above that of reference plots using proportional integrative derivative (PID) control have been reported for several ecosystems including grass (Nijs et al., 1996; Van Peer, Nijs, Reheul, & De Cauwer, 2004), tundra (Marchand, Mertens, Kockelbergh, Beyens, & Nijs, 2005; Nijs et al., 2000), grazing land (LeCain et al., 2015; Luo et al., 2010), wheat fields (Kimball et al., 2015; Wall, Kimball, White, & Ottman, 2011), paddy rice (Gaihre et al., 2014; Rehmani et al., 2011), soybean (Ruiz‐Vera et al., 2013; Siebers et al., 2015), and maize (Ruiz‐Vera, Siebers, Drag, Ort, & Bernacchi, 2015), as well as tree saplings (McDaniel et al., 2014; Rich et al., 2015). However, all of these studies had 3‐m‐diameter plots or smaller, and most warmed by only 1.5°C during daytime. Although short‐term heat‐wave simulations have achieved higher degrees of warming (e.g., De Boeck, Dreesen, Janssens, & Nijs, 2011; Marchand et al., 2005; Siebers et al., 2015; Van Peer et al., 2004), the highest reported season‐long warming of a 3‐m‐diameter plot is 3.5°C (Ruiz‐Vera et al., 2015). Our experimental system expanded plot size to 4 m diameter and the degrees of warming to 4°C. The projected warming in Puerto Rico is greater than the global averages (Henareh Khalyani et al., 2016).

Further, we adapted an IR heater system for a wet tropical forest with high humidity, hurricanes, power surges and outages, clayey soils, and a steep slope. The solutions described herein address these challenges to warming tropical forest understory plants and soils and provide valuable information for designing future warming experiments for taller vegetation in a number of underrepresented ecosystem types.

## 2 MATERIALS AND METHODS

### 2.1 Site

**Figure 1:**
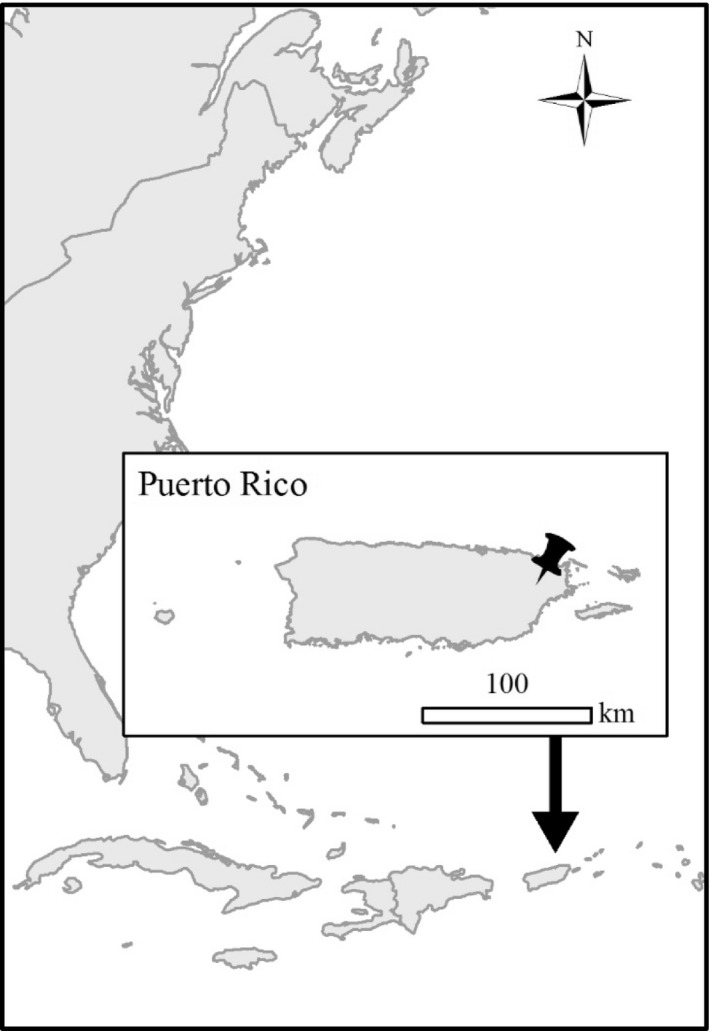
Geographic site map showing location of Puerto Rico with respect to continental United States and insert showing location of USDA Forest Service Sabana Field Research Station within Puerto Rico

**Figure 2:**
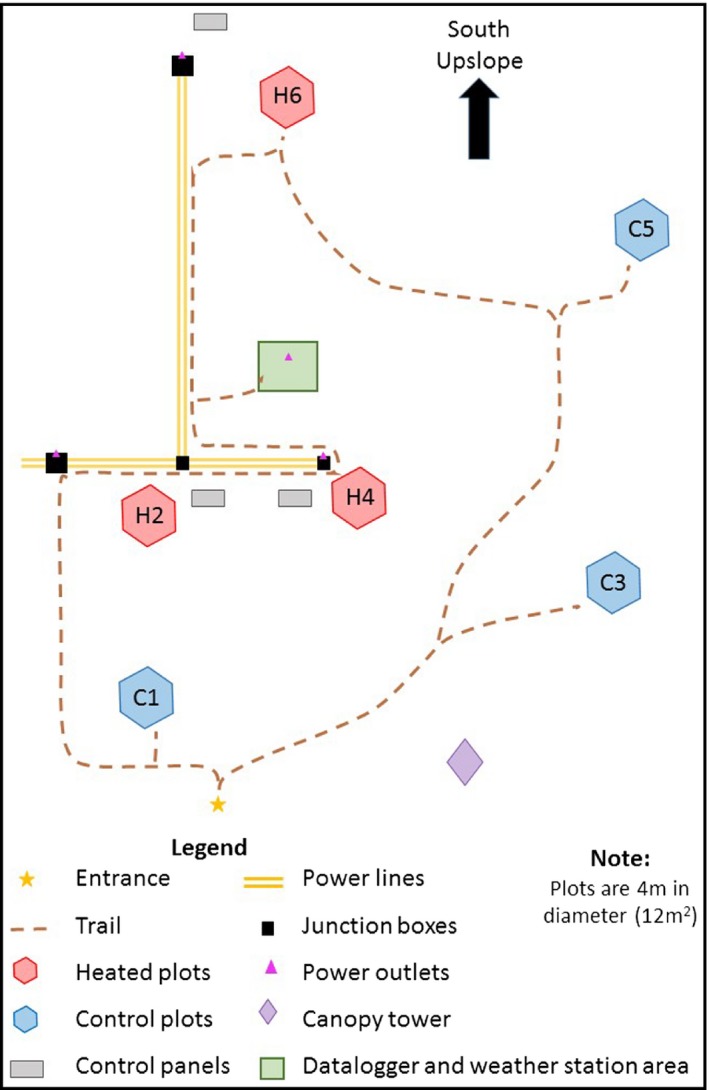
Schematic plan to scale showing layout of plots in the Luquillo Experimental Forest

The experimental site is located in northeastern Puerto Rico near the USDA Forest Service Sabana Field Research Station in the Luquillo Experimental Forest (LEF; 18°18′N, 65°50′W) (Figures 1 and 2). The site is classified as a subtropical wet forest according to the Holdridge Life Zone System (Holdridge, 1967) and is a secondary forest, regenerated naturally from pasture since the early 1950s. Mean annual temperature is 24°C, with month‐to‐month variation of just 4°C, and mean annual rainfall is 3,500 mm (García‐Martino, Warner, Scatena, & Civco, 1996). While the driest period generally occurs from January to March, there is no significant “dry season,” as every month receives greater than 200 mm and precipitation is highly variable (Heartsill‐Scalley, Scatena, Estrada, McDowell, & Lugo, 2007). Located at 100 m elevation, the site is characterized by relatively steep slopes ranging from 15 to 26°, with an average slope of 21° (Table 1). Soils are classified as Ultisols and are deep, clay‐rich and acidic, with high aluminum and iron content (Scatena, 1989). Root biomass is concentrated (>60%) in the top 10 cm of mineral soil (Silver & Vogt, 1993).

**Table 1:** Slopes of the soil surface in the various plots of the TRACE experiment (See Figure 2 for plot locations)

Plot	Slope (°)
C1	20
H2	26
C3	15
H4	18
C5	16
H6	18
Weather Station	22

### 2.2 Equipment

**Figure 3:**
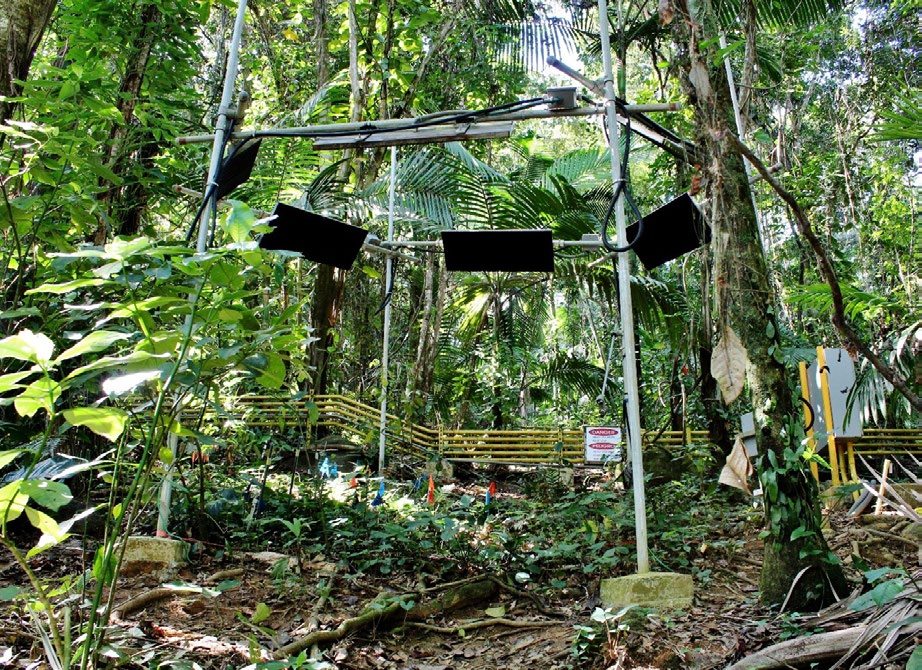
Photograph of a heated plot showing the understory vegetation beneath the tree canopy along with six infrared heaters deployed on pipes arranged in a hexagon. The vertical pipes are posts set in concrete footings, two of which are visible in the foreground. The gray control panel is to the right. In the background is the 480 V, 3‐phase power line in yellow conduit. (Photo by Mónica Cruz)

The basic design of the IR heater system was that of the T‐FACE (temperature free‐air controlled enhancement) described by Kimball et al. (2008), which produced uniform warming across hexagonal plots with six IR heaters (Figure 3), and which was able to maintain a constant temperature rise of the heated plots above that of control or reference plots for growing‐season‐long periods of time. In our case, the slope (~21°; Table 1), saturated soils, and high clay content provided additional challenges. Galvanized 38 mm steel pipes (nominal 1½ inches) were installed to 0.7 m depth in 0.3 m × 0.8 m concrete footings for each of the six hexagon points. The footings and posts with attached cross‐bars provided stable structures and prevented slippage down slope. The heaters were bolted to cross‐bars (38 mm galvanized steel pipes), which spanned the space between posts on the periphery, and which enabled the heaters to face the center of the plots. The cross‐bars were attached to the vertical posts with Kee Klamps (Kee Safety Inc., Buffalo, NY), allowing heaters to be mounted at the same height above the vegetation regardless of slope. The upslope and downslope heaters were deployed with long axes horizontal, while the long axes of the side heaters were parallel to the slope. The short axes of all heaters were tilted 45° with respect to their vertical support posts. The variability of the height of the vegetation provided another challenge, so heaters were mounted at 0.4 times plot diameter above three‐fourth the height of the tallest plants to provide as uniform warming as possible without exposing the tallest vegetation to too much heat. A small junction box was installed for each heater and connected to a larger junction box for each heated plot via a waterproof, flexible “super service” cable. This cable along with the use of Kee Klamps enabled the heaters to be raised and lowered for repairs and to adjust for changing vegetation heights.

As specified by a civil engineer, the concrete footings, posts, and cross‐bars with the heaters should be able to withstand hurricane‐force winds with the heaters tilted vertical at a maximum operating height of 3.6 m with no allowance for a windbreak from the trees. Nevertheless, when a hurricane is forecast, the plan is to shut off the system and lower the heaters to just above vegetation height. During the time this study was undergoing journal review (September 2017), Hurricanes Irma and Maria struck Puerto Rico, including the experimental site. Heaters were lowered in advance of the stronger Hurricane Maria but not for Irma. The structures withstood the winds of both hurricanes. However, falling trees and branches, for which there is no protection, damaged portions of the plot infrastructure, the majority of which was to the supporting posts for the heaters. Based on our observations from the two storms, the infrastructure damage from fallen trees was more substantial when the heaters were not lowered. Nevertheless, all damage is repairable, and we expect the experiment will be operational again by the time power is restored to the area. Thus, there is promise for the design even in areas where disturbances such as hurricanes are common.

Based on theory (Kimball, 2005) and experience (Kimball et al., 2008) and the weather data available at the design stage, larger heaters were required to warm 4‐m‐diameter plots than had been used previously in such experiments. The selected heaters were rectangular black‐body radiators (Model Raymax 1010, Watlow Electric Manufacturing Co., St. Louis, MO [currently manufactured by Heat and Sensor Technology, Lebanon, OH], 1,143 mm × 457 mm × 47.4 mm, 8,100 W, 480 V, 3‐phase). They were constructed of stainless steel and welded around the edges to be liquid tight, with insulation behind the internal electrical heating wires to minimize heat loss from the back. We requested that the welded lip be around the hot front side because in our application they are pointed downwards, and we wanted to minimize leaves and other debris being caught by the lip. The emitting face was painted black, and the maximum watt density of 15.4 W/m^2^ ensures that they emit almost no radiation at wavelengths <850 nm, which may be photochromatically active in plants (Kimball, 2005; Kimball, Conley, & Lewin, 2012). The three control or reference plots had identical posts and cross‐bars to the three heated plots, but with dummy heaters made of sheet metal the same size as the real heaters and painted black on one side. The unheated control plots had no electrical power cabling.

**Figure 4:**
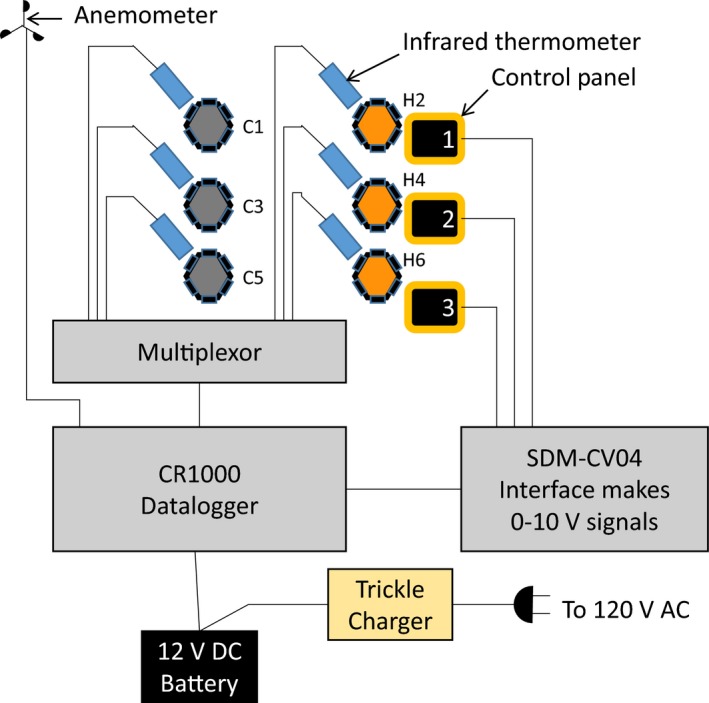
Schematic diagram of components to control the temperature rise of heated (orange) hexagonal plots above the temperature of corresponding reference or control (green) plots. Additional weather instruments are also connected to the datalogger

The PID control system was that of Kimball (2005), adapted for newer equipment (Figure 4). Understory vegetation temperatures were continually measured in all plots using IR thermometers (Model SI‐121, Apogee Instruments, Logan, UT). A datalogger plus multiplexor (Model CR‐1000 logger with Loggernet software and Model AM16/32 multiplexor, Campbell Scientific, Logan, UT) contained the PID logic to maintain the desired 4°C difference between the heated and control plots. The logger sent signals to an interface (Model SDM‐CV04, Campbell Scientific), which generated a 0–10 V signal to control the AC voltage supplied to the heaters. The vegetation temperatures from heated plots were adjusted for IR radiation from the IR heaters that was reflected from the plots and sensed by the IR thermometers within their 8–14 μm waveband (Kimball et al., 2008). The amount of reflected radiation *R*_*f*_ (W/m^2^) was calculated from:

(1) $$R_{f}=R_{H}\times {\left(\text{PID volts}/10\;V\right)}^{2}\times f_{8-14}\times \rho \times \eta$$

where *R*_*H*_ is the heater capacity (W/m^2^), *f*_8–14_ is the fraction in the 8–14 μm waveband (assumed 0.13), ρ is the vegetation surface reflectance (assumed 0.02), and η is the heater array efficiency computed from:

(2) $$\eta =0.10+0.25e^{\left(-0.17u\right)}$$

where *u* (m/s) is wind speed (Kimball & Conley, 2009; Kimball et al., 2008). To account for stochastic events (e.g., falling palm fronds) and random temperature spiking in individual control plots (e.g., from sunflecks), the vegetation temperatures from the control plots were averaged, and then PID control signals were computed for the individual heated plots from the differences between the individual vegetation temperatures of the heated plots and the average of the control plots.

**Figure 5:**
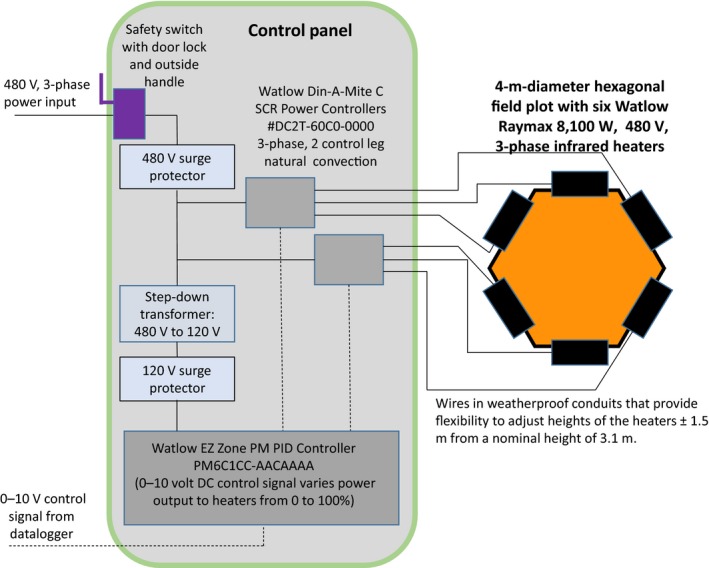
Schematic diagram of a control panel

**Figure 6:**
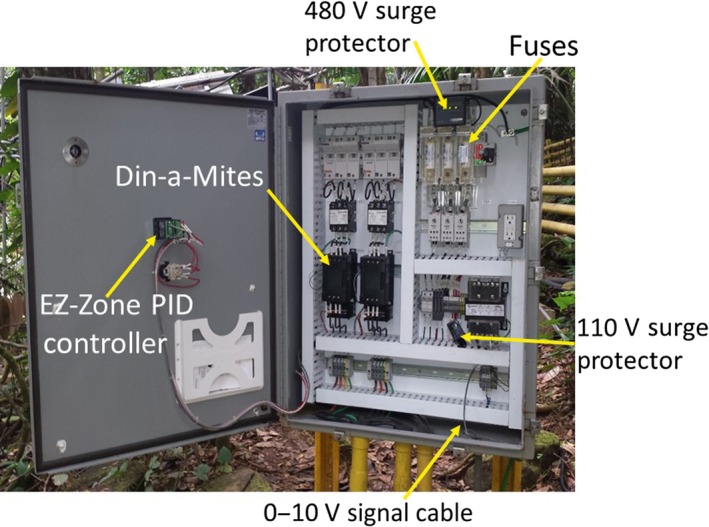
Photograph of actual control panel with major components labeled. (Photo by Aura M. Alonso‐Rodríguez)

The control panels, designed and constructed at Watlow Electric Manufacturing Co., contained Din‐a‐Mite C SCR power controllers (Model #DC2T‐60C0‐0000, 3‐phase, 2 control leg, natural convection). Each had the capacity to modulate the voltage for three heaters (Figures 5 and 6). Thus, two Din‐a‐Mites were required per plot. One EZ Zone PM PID Controller (Model PM6C1CC‐AACAAAA) provided an interface between 0 and 10 V signals coming from the SDM‐CV04 and the Din‐a‐Mites. The EZ Zone controller operated with 120 V AC power, so a step‐down transformer was needed to connect it to the 480 V line power. Unfortunately, with our tropical conditions and frequent power surges, we soon learned that, in addition to factory‐supplied fuses, a 600 V surge protector for the main power line with three 480 V surge protectors for each plot were needed, as well as 480 V surge protectors where the line power entered each panel and another 120 V surge protector for the power going to the EZ Zone controller. To ensure a safe working environment in this high voltage system, we grounded each control panel, as well as every steel post of all hexagonal plots, the primary junction boxes for each warmed plot, and all dataloggers.

High humidity resulted in components rusting, growing mold, and creating an environment with high potential for arc flash and electrical shorts. We used high heat Rustoleum paint to seal any cuts made to the steel posts to deter rust. The control panel casing was made of painted steel. We added additional sealing to the doors of each of the control panels to reduce humidity entering the casing. We also implemented a regular 3‐month scheduled shut‐down, examination, and maintenance of the internal components of the control panels with a certified electrician and a 6‐month inspection of electrical connections, cabling, and grounding. Finally, we added code to the control program in the datalogger to prevent the PID signal from dropping below 0.05 V, which keeps the heaters and other equipment at a minimum of 0.5% of capacity to maintain enough residual warmth for the components inside the control panels to stay dry as long as the 480 V power is on.

A weather mast was installed near the middle of the experimental area to collect understory microclimatic data (Figure 2). Instruments included a cup anemometer and wind vane (Model 03002‐L, Campbell Scientific), pyranometer and quantum sensors (Models SP‐110 and SQ‐110, respectively, Apogee Instruments), and an air temperature and humidity probe (Model CS215, Campbell Scientific), all connected to the same datalogger used to control the IR heater system. Weather, canopy temperature, and heater control data were output as hourly averages. The wind and air sensors were deployed at 3.0 m above the soil surface, and the radiation sensors at about 3.5 m. A thermocouple to measure the surface temperature of one of the heaters in Plot H4 was also connected. Rainfall data presented herein came from a tipping bucket rain gauge (Model TR‐525‐W2; Texas Electronics, Inc.; Dallas, TX USA) on a standard weather station in the open about 200 m from the experimental site.

Five soil moisture/temperature probes (Model CS655, Campbell Scientific) were installed in each of the six plots and recorded hourly. Three probes were installed at the 0–10 cm depth: one at the center of each plot, a second at the edge of plot, and a third midway between center and edge. The fourth and fifth probes were installed at the 20–30 and 40–50 cm depths, respectively, at the center of the plots. The deeper probes were installed in a single 5‐cm‐diameter hole at a 45° angle into the soil wall.

Power lines to the field site were conveyed in above‐ground electrical conduits with concrete supports at about 1.5‐m intervals (Figures 2 and 3). Power lines were above‐ground to minimize disturbance to the root and soil system of the study site, to facilitate regular maintenance of the junction boxes, and to facilitate removal when the project is terminated. At each of the power line junction boxes associated with the three control panels, we included 120 V power outlets to accommodate scientific equipment and small tools (Figure 2). Twelve‐volt‐DC sensors and dataloggers were connected to a marine battery, which in turn was connected to a 120 V AC trickle charger and a backup generator. Therefore, data collection continued during power outages and system maintenance, even when there was no power supply to the heaters. The battery also provided additional lightning protection to the logger and sensors.

### 2.3 Actual and theoretical power use calculations

For six 8,100 W heaters deployed over a 4‐m‐diameter plot, if the system were 100% efficient, the available heating capacity would be 3,867 W/m^2^. By multiplying 3,867 times the PID signal (divided by 10) squared and then dividing by the measured temperature rise, the actual average hourly power use per degree of temperature rise for each heated plot was obtained.

The theoretical average hourly power use per degree of temperature rise for each heated plot was calculated following Kimball (2005). First, the IR heating requirement was calculated, which required a value for canopy resistance. Measurements of stomatal conductance on the understory vegetation produced an average value of about 0.038 mol m^−2^ s^−1^ (equivalent to 1,000 s/m stomatal resistance; data not shown), which is very low compared to most vegetation that is adapted to growing in full sunlight. Leaf area index was estimated to be about 0.5, and the soil was covered with dead leaves that were dry except when covered with dew or rain. Therefore, canopy resistance was estimated to also be about 1,000 s/m. However, the IR heating requirement proved quite insensitive to the estimate of canopy resistance due to the very humid conditions with low evapotranspiration. In contrast, there was more uncertainty in the aerodynamic resistance introduced by the high stall speed (0.5 m/s) of the anemometer and the calm understory conditions. Under stall conditions, wind speed was assumed to be 0.25 m/s. Next, the radiative efficiency of the heaters was calculated following Kimball (2005), and again the poor wind measurements under the very calm conditions introduced uncertainty. Finally, the theoretical IR heater requirement was divided by the radiative efficiency and the geometric efficiency for a hexagonal IR heater array (0.372 from Kimball et al., 2012) to obtain the theoretical average hourly power use per degree of temperature rise.

## 3 RESULTS AND DISCUSSION

### 3.1 Uniformity of warming

**Figure 7:**
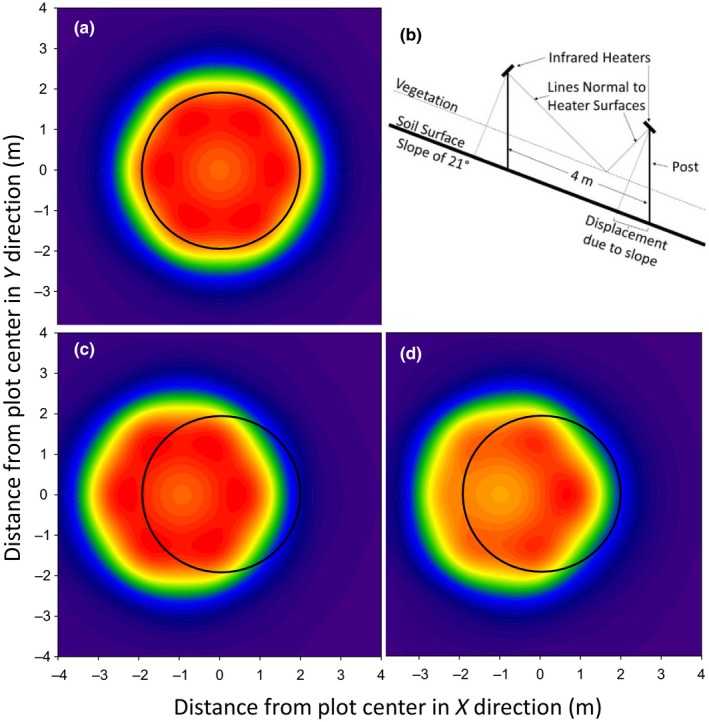
(a) Theoretical distribution of infrared radiation at the top of vegetation surface from six heaters deployed in a hexagonal pattern around a 4 m‐diameter plot with no slope at a height of 1.43 m above the vegetation, pointed toward the center, and tilted 45° from horizontal (following Kimball et al., 2012). (b) Schematic diagram illustrating the average 21° slope of the heated plots, the vertical posts, and the 45° tilt of infrared heaters with respect to the posts at the top and bottom of the plots. (c) Like (a) but for a sloped soil surface with 1‐m‐tall vegetation and the heaters tilted 45° with respect to the soil surface. The radiation is displaced upward (d). Like (c) but the heaters are tilted by 45° with respect to their posts as illustrated in (b). Some of the radiation is still outside the plot on the upslope side, but much more is distributed across the plots

**Figure 8:**
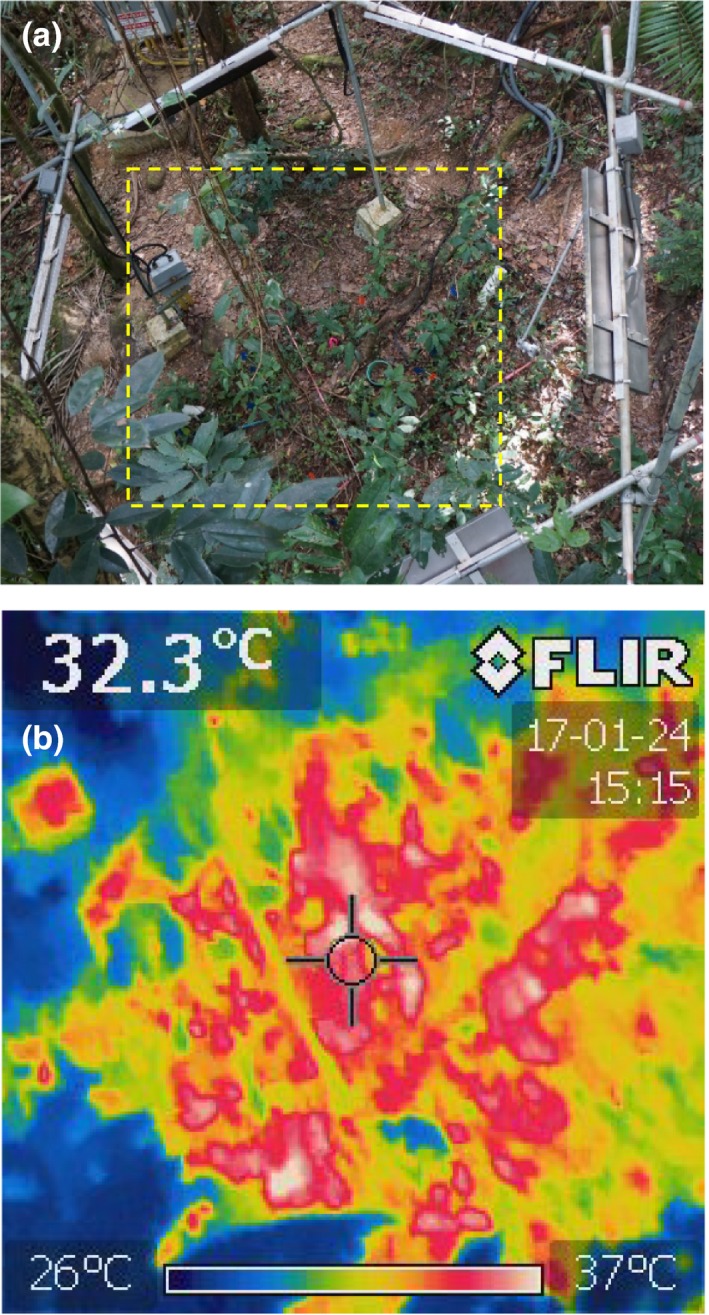
Top. Photograph of heated plot (H4) from a ladder leaning against a tree near the plot at a height of about 7 m above the ground. The square with dashed outline shows the approximate view of the thermal imager used to obtain the bottom thermal image from the same ladder position. For orientation, the rectangular hot spot at the left edge about 1/3 of the way down from the top was the electrical junction box, which appears gray in the photograph. The linear cooler stripe that extends from upper left downward to the right is due to a vine hanging from a branch high over the plot down to the soil surface. A somewhat similar cool stripe from upper right that extends downward toward the left is due to several plants in a row. The very cool leaves at the bottom left are actually above the height of the heaters (not considered understory) and are not being warmed. (photograph and image taken by Bruce Kimball)

Theoretically, a hexagonal array of IR heaters should produce uniform warming across a circular plot (Figure 7a). However, the understory vegetation in this experiment was very nonuniform, with varying height and ground coverage (Figures 3 and 8a). Consequently, the warming also appears very nonuniform (Figure 8b). Closer comparison between Figure 8a and b, however, revealed that the hot spots corresponded to areas where dry leaves on the soil surface were visible. The areas of green vegetation, at which the IR thermometers were pointed, were being warmed close to the target +4°C. The warming of live foliage was therefore relatively uniform, and thus, the deployment heights and angles of the six heaters were able to provide consistent warming to the understory vegetation.

The slope of the site also affected the IR radiation distribution. Hexagonal heater arrays theoretically and actually can produce uniform warming on sites with zero slope (Figure 7a; Kimball et al., 2008, 2012), and if the posts were installed at an angle from vertical equal to the slope, a distribution like 7a should be possible on sloped plots. However, it is not easy to bore holes in the soil, pour concrete, and set posts at nonvertical angles, and then, the resultant structure would have a constant torque, so our posts were installed vertically. If the heaters are tilted at 45° with respect to a sloped soil surface and the heater posts are vertical, theoretically the area of uniform radiation would be displaced upslope (Figure 7c). In our case, we tilted the heaters at 45° with respect to their vertical posts (Figure 7b), rather than the soil surface, which theoretically distributed the warming more evenly over the plots, although some radiation still falls outside the plot area on the high side while the downslope edge of the plot receives less (Figure 7d). Nevertheless, the theoretical proportion of total radiation falling within the plot was only reduced to 36% compared 39% for level plots.

**Figure 9:**
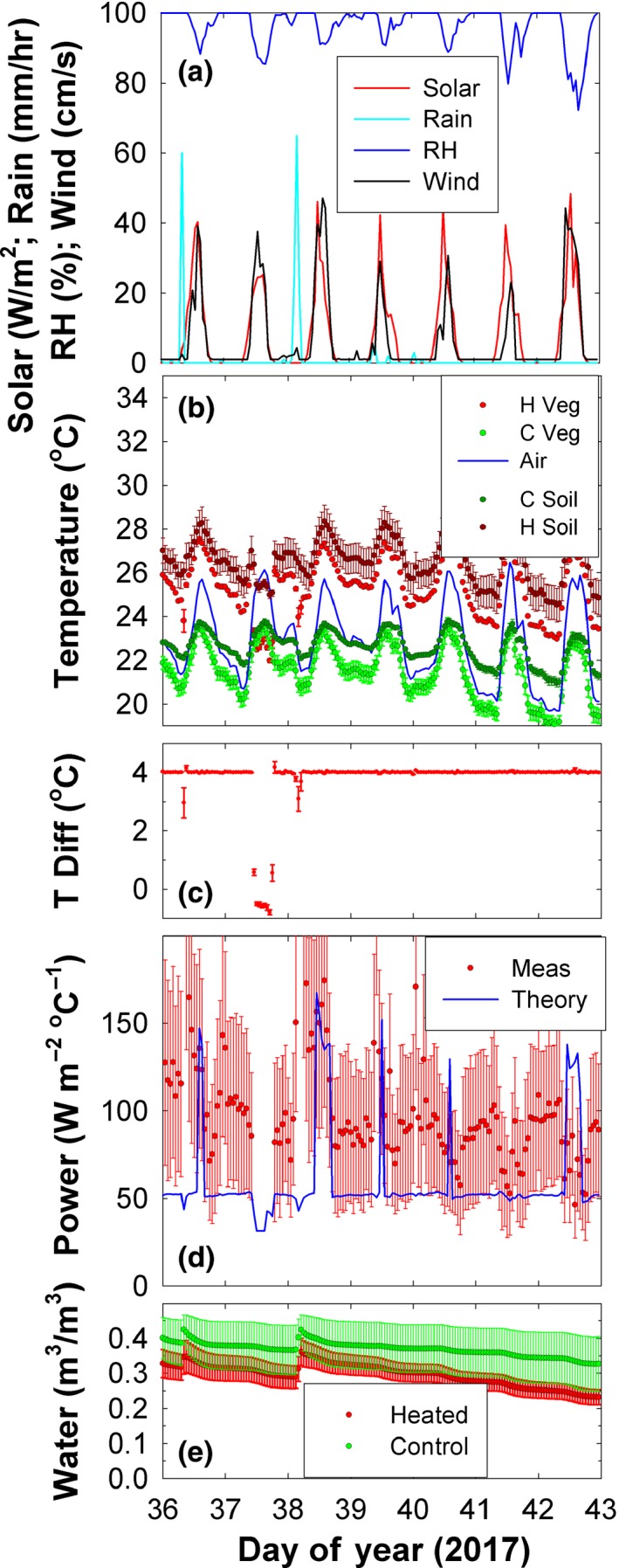
Infrared heater system performance during the week starting on day 36 of 2017 (130 days after the start of the warming treatment). (a) Solar radiation, rainfall (in the open), relative humidity, and wind speed. (b) Heated and control understory vegetation temperatures (± standard errors), air temperatures, and heated and control soil temperatures at the 0–10 cm depth at the center of the plots. (c) Heated minus control understory vegetation temperatures. (d) Measured power consumption and that following the theoretical equations of Kimball (2005). (e) Heated and control soil water contents at the 0–10 cm depth in the center of the plots

**Figure 10:**
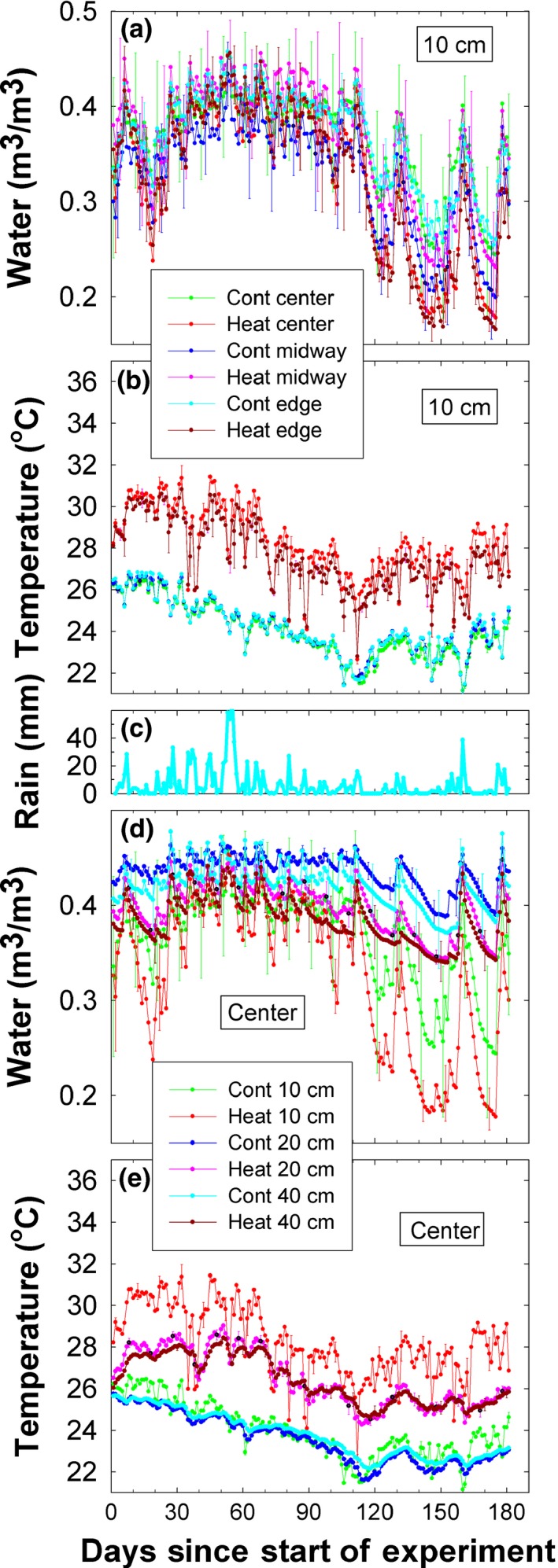
Soil water contents and soil temperatures during the first 6 months of the experiment in the control (Cont) and heated (Heat) plots. (a) Hourly average soil water contents for 0–10 cm depths at the centers of the 4‐m‐diameter plots, at midway between plot centers and edges, and at the edges of the plots. The values are the means of three replicates observed at 13:00 each day. Standard errors are shown, but only for every 10 days to improve clarity. (b) Same as (a) but for soil temperatures. (c) Daily rainfall observed in the open about 200 m from the plots. (d) Soil water contents similar to (a), but measured in the centers of the plots at the 0–10 cm depths, at the 20–30 cm depths, and at the 40–50 cm depths. (e) Soil temperatures similar to (b), but measured in the centers of the plots at the 0–10 cm depths, at the 20–30 cm depths, and at the 40–50 cm depths

**Figure 11:**
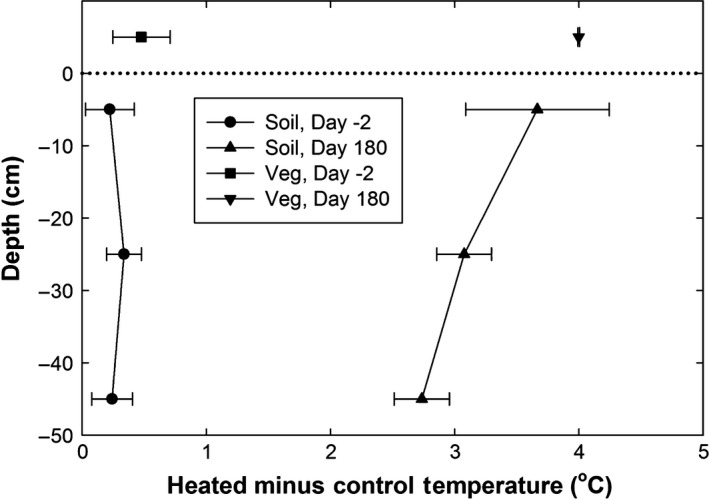
Profiles of the differences in soil temperature between heated and control plots at the 0–10, 20–30, and 40–50 cm depths that were observed at midnight 2 days before and 180 days after the start of the 4°C IR heating treatment. The sensors were at the centers of the 4‐m‐diameter plots. Also shown are the corresponding differences in vegetation canopy temperatures, which have been plotted at a height of 5 cm for convenience

As expected, the IR heater array warmed the shallow soil (Figures 9b, 10b,e and 11). At the 0–10 cm depth in the center of the heated plots, soil temperatures were ~1°C warmer than the understory vegetation temperatures at night, with less of a differential at midday (Figure 9b). Under control conditions, there was very little spatial variability of the 0–10 cm soil temperatures across the plots from center to edge (Figure 10b). The plot center position in the heated plots, however, was slightly hotter (<1°C) than either edge or midway (between center and edge) positions (Figure 10b). Moreover, the IR system consistently maintained the 4°C target temperature rise in surface soils during the whole season, except when there were power outages, as evidenced by the downward spikes in the curves for the heated plots (Figure 10b).

The temperature of the surface soils (0–10 cm) of the heated plots rose to approximately 3.6°C warmer than the control plots within 6 days of the start of warming. In contrast, the soil temperatures at both 20–30 and 40–50 cm depths were very similar to those of the control plots at the start of the experiment (Figures 10e and 11), but the temperature difference steadily increased for about a month, leveling off at approximately +3°C warming at 20–30 and 40–50 cm depths, respectively, for the remaining observation period (Figures 10e and 11). Averaged over the whole six months, the averaged warming was 2.9 and 2.6°C for the two depth increments, respectively (Table 2). Soil temperatures at deeper depths were less responsive to the power outages, but the overall average warming may have been greater if there had been no outages. There were no sensors below 10 cm at the edge of the plots, so we cannot be sure the warming at depth in the centers extended out to the edges. However, unlike open‐top or other chambers, the warming from IR heaters generally extend outside the defined plot area (Figures 7 and 8). For a hexagonal array, 63% of the IR radiation theoretically falls outside the plot area (Kimball et al., 2012), warming the soil under the plot edges and serving as a buffer around the periphery. Overall, the consistent warming of surface soils to 3.6°C and the loss of just 1°C warming with depth demonstrates a strong heat holding capacity of these clayey soils, despite the high rainfall environment. Taken together, these findings suggest this IR warming design can be utilized to examine the effects of warming on soil processes as deep as 50 cm, if not more.

**Table 2:** Soil temperature (T) and volumetric soil moisture contents (M) for 0–10, 20–30, and 40–50 depth increments in the control and heated plots that were averaged over a year before the start of the warming treatment and for 6 months after the start of the treatment

Soil depth (cm)	Variable	Pretreatment (~1 year)				Treatment (~6 months)			
		Control		Heated		Control		Heated	
		Mean ± *SE*	Range	Mean ± *SE*	Range	Mean ± *SE*	Range	Mean ± *SE*	Range
0–10	T (°C)	24.3 ± 0.08	9.15	24.3 ± 0.07	8.90	23.1 ± 0.06	8.22	26.4 ± 0.32	10.31
20–30	T (°C)	25.1 ± 0.01	3.05	25.3 ± 0.01	3.36	23.6 ± 0.02	4.22	26.5 ± 0.02	5.16
40–50	T (°C)	25.1 ± 0.01	2.56	25.2 ± 0.01	2.78	23.7 ± 0.02	3.53	26.3 ± 0.02	4.11
0–10	M (m^3^/m^3^)	0.32 ± 0.0004	0.27	0.36 ± 0.0006	0.27	0.35 ± 0.0008	0.26	0.35 ± 0.001	0.28
20–30	M (m^3^/m^3^)	0.43 ± 0.0002	0.08	0.42 ± 0.0003	0.11	0.44 ± 0.0003	0.10	0.40 ± 0.0004	0.13
40–50	M (m^3^/m^3^)	0.42 ± 0.0003	0.11	0.40 ± 0.0004	0.13	0.42 ± 0.0003	0.12	0.39 ± 0.0004	0.14

### 3.2 Effects on soil moisture

As expected, the warming treatment resulted in a stronger dry‐down of the soil of the heated plots than in the control plots during periods of low rain, especially in the shallow soil layer (Figures 9e and 10a,d). However, the high rainfall environment led to rapid re‐wetting with overall no difference in average soil moisture values between heated and control plots. Although soil moisture was much more temporally variable than temperature (Figure 10b), there appeared to be little or no spatial variation in soil water content from the centers of the plots to the edges (Figure 10a). Water contents at the deeper depths were greater and fluctuated less dramatically than those at 0–10 cm (Figure 10d). From about Day 20 through Day 100, there was ample rain (Figure 10c), and consequently, the effects of heating on soil water content were not as pronounced (Figure 10a,d). However, there were four distinct drying periods during which the heated plots dried faster and more than the controls, peaking around 20, 125, 145, and 175 days from the start of the experiment (Figure 10d). Nevertheless, the average soil moisture remained adequate for the plants. While there were stronger drying events in the heated plots, over the course of 6 months of the warming treatment, the differences in mean soil moisture between the control and heated plots were not different in the surface soils (0.35 m^3^/m^3^; Table 2). The observed drying effect was overall stronger in the deeper, more buffered soils, with the heated plots approximately 0.036/m^3^/m^3^ drier than the control plots at both 20–30 cm and 40–50 cm depths.

It is not surprising that the warming treatment caused more soil drying. The IR warming system warms foliage and soil surface, but does not directly affect the absolute humidity of the air. Some observations and global circulation models suggest that relative humidity, not absolute, will remain more or less constant with global warming (Dessler & Sherwood, 2009). On the other hand, more recent calculations by Sherwood and Fu (2014) suggest that, in contrast to the whole earth with land and ocean surfaces, global warming over the continental land surfaces is likely to lead to overall drying, that is, a reduction in relative humidity and closer to warming at constant absolute humidity. Thus, the T‐FACE system likely produced conditions similar to those expected with future global climate change.

### 3.3 Reliability and Power Issues

The primary obstacle to having a highly reliable warming system was the unreliability of the main power supply to the station, which could be a common issue at numerous sites around the world. During the first 6 months of operation from 28 September 2016 to 28 March 2017 (4,350 total hours), the heaters were off 10% of the total time due to power outages. For the safety of personnel, the 480 V power to individual plots was turned off when individuals were working in the plots, amounting to another 1% of the total off time in the first 6 months. Fortunately, after power outages, the system rapidly re‐equilibrated once power was restored. For example, there was a power outage for about 6 hr on the afternoon of DOY 37 (Figure 9). During these hours, the temperatures of the heated plots dropped close to those of the control plots (Figure 9b). After power was restored, the 4°C target was achieved within an hour.

During the first 6 months, four of the 18 heaters failed and were replaced. Condensate may have formed during a power outage, resulting in a short circuit when power was restored. Three of the heaters were in the same plot, so alternatively perhaps there was a lightning strike near this plot.

### 3.4 Performance

**Figure 12:**
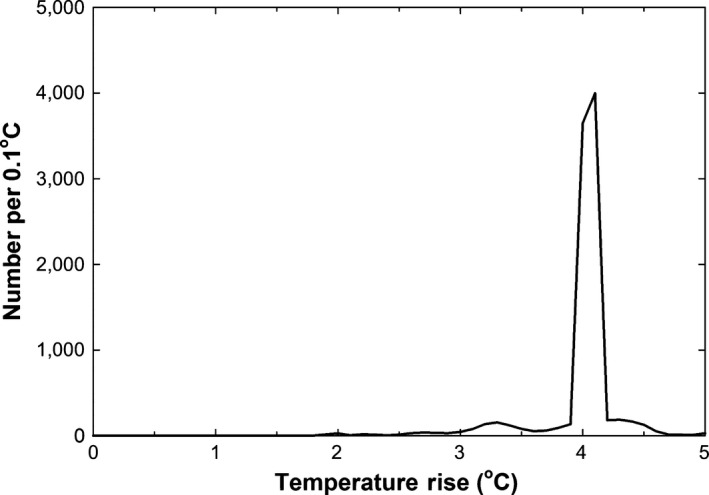
Histogram of hourly average understory vegetation temperature differences between heated and control plots between 28 September 2016 and 28 March 2017. Individual replicates were kept separate. Hours when there were power outages were omitted. The total number of observations is 9,651

The forest understory microclimate was dark, humid, and calm (Figure 9a). Solar radiation was an order of magnitude lower than out in the open. Relative humidity was generally above 80% even at midday and 100% at night. Wind speeds were very low, often below the stall speed of the anemometer. Under these calm and humid conditions, the IR heater system successfully maintained the +4°C target increase of the understory vegetation temperatures (Figures 9b,c and 12). Although there were minute‐to‐minute variations of the temperature rise with gusts of wind and passing clouds, the hourly average temperature rise was consistently within 0.1°C of the 4°C target (omitting hours with power outages) during the first 6 months of operation (Figure 12), which is excellent performance. The heaters were below 35% of capacity 95% of the time, so for this tropical forest understory site, we could have tripled plot areas or used equivalently smaller heaters.

Surprisingly, the system maintained relatively constant heating under rainy conditions. For example, 11 mm of rain was received on DOY 38, enough to significantly increase soil moisture at the 10 cm depth (Figure 9e), yet the temperature rise of the heated plots dropped only one degree below the 4°C target (Figure 9b,c).

The actual measured power consumption per degree of temperature rise was generally only 100 W m^−2^ °C^−1^ (Figure 9d), which is somewhat low, but consistent with the calm, humid conditions. The theoretical curve is generally lower than the measured values but near the lower error bar values, which again gives credence to the theoretical equations that can be used to aid in the design of future experiments.

## 4 CONCLUSIONS AND IMPLICATIONS

For millennia, tropical forests have persisted under a relatively stable thermal environment; yet, in the next 80 years, climate change is expected to increase global temperatures by up to 4°C (IPCC, 2014). Because tropical species are adapted to very narrow thermal niches, their ability to tolerate and acclimate to increased temperatures may be much more limited than in higher‐latitude ecosystems (Janzen, 1967). If true, relatively small increases in temperature could have very large consequences for the substantial amounts of carbon cycled by tropical forests. Until now, logistical challenges such as high rainfall and humidity and inconsistent power have meant that the field warming experiments, which have been so influential in our understanding of ecosystem responses to warming in other biomes, have been absent in tropical forests. Here, we describe solutions developed in response to these unique challenges, providing a significant step forward in our ability to experimentally warm a wider range of ecosystem types.

As a whole, the IR Heater system maintained the target 4°C rise in temperature of the heated plots above that of the controls when power was available (Figure 12), and uniformity of the treatment effect was satisfactory (Figure 8). Furthermore, the system achieved significant soil warming of 2.6°C on average to a depth of 50 cm with minimal soil drying. To achieve this success, several obstacles were addressed (Table 3). Thus, based on our experience reported herein and that of many prior experiments listed in the Introduction, our system can be used to study tropical and nontropical forest understory plants and soils, as well as agricultural crops and rangeland, up to a vegetation height of at least 1.3 m (one‐third the plot diameter) and a soil depth of approximately 50 cm (one‐eighth plot diameter).

**Table 3:** Summary of the problems encountered at this site different from those of previous infrared (IR) warming experiments and the solutions used to overcome them

Problem	Solution
Power surges and outages	600 V surge protector at main power line.
	480 V surge protectors in the main circuit breaker.
	480 V surge protector where power line enters the control panel.
	120 V surge protector where the power goes to the EZ Zone controller.
	Backup battery and generator power allowed data collection to occur during power outages.
High humidity	Liquid tight welding.
	Rustoleum paint to seal any cuts made to the steel posts.
	Control panel casing made of painted steel.
	Added additional sealing to the doors of each control panel.
	Implemented a scheduled shut‐down every 3‐months to examine the internal components of the control panels and provide maintenance as needed.
	Added code to the control program so the PID signal could not drop below 0.05 V, allowing the components inside the control panels to maintain enough residual warmth to stay dry.
Steep slope with weak soils when wet	Tilt of heaters 45° with respect to vertical posts (rather than soil surface).
	Vertical posts set in concrete.
Hurricanes	Civil engineer‐specified design of concrete footings for the posts to withstand code design winds for the region with the heaters tilted vertical at maximum operating height (3.6 m) with no allowance for windbreak effects of the trees [withstood Hurricane Irma and Maria winds (but not falling trees)].
	Movable cross‐bars to lower during hurricane.
	Easy power off switch at the main circuit breaker.
Heterogeneous understory	Heater height set at 0.4 times plot diameter above three‐fourth of the height of the tallest plants. The height was applied with respect to the soil surface at the bottom and top of the plots accounting for slope.
Vegetation above heaters	The welded lip of each IR lamp was placed around the hot front side, which was pointed downwards to minimize leaves and other debris being caught by the lip.

Tackling the larger issue of the effects of global warming on whole forest ecosystems and their climate feedbacks would ideally be achieved with larger plots with adult trees. Here, we successfully extended both plot size and degrees of warming with heaters 4–8 times larger than previously used, which were formerly untested for this application. No scaling issues were encountered, with relatively uniform warming of vegetation and soils of 4‐m‐diameter plots, so it should be feasible to combine hexagonal arrays into honeycombs as suggested by Kimball et al. (2012) and extrapolate to 30‐m‐tall trees and the 100‐m scale plots needed for forest and orchard experiments.

## CONFLICT OF INTEREST

None declared.

## AUTHOR CONTRIBUTIONS

TW, MC, and SR involved in conceptualizing and planning experiment; BK, TW, and GG involved in design of warming system and power infrastructure; TW, BK, GG, and AA involved in construction and installation of heater system and power upgrades; TW and AA performed maintenance of experiment; SR, MC, TW, and AA performed installation of soil and plant sensors and making measurements; all involved in writing and editing the manuscript.
